# Survey of doctors’ perception of professional values

**DOI:** 10.1371/journal.pone.0244303

**Published:** 2020-12-28

**Authors:** Nadia Minicuci, Cinzia Giorato, Ilaria Rocco, Peter Lloyd-Sherlock, Giampiero Avruscio, Fabrizio Cardin

**Affiliations:** 1 National Research Council, Neuroscience Institute, Padova, Italy; 2 Department of Surgery Oncology and Gastroenterology, Padova, Italy; 3 School of International Development, University of East Anglia, Norwich, United Kingdom; 4 Department of Cardiac, Angiology Unit, Thoracic and Vascular Sciences, University Hospital of Padua, Padua, Italy; Hackensack University Medical Center, UNITED STATES

## Abstract

**Background:**

New challenges in the medical field of the third millennium emphasise the "humanization of medicine" leading to a redefinition of doctors’ values, limits and roles. The study aims to assess whether there are different personality dimensions of physicians in relation to their perception of professional values and public expectations.

**Methods:**

A questionnaire on the perception of professional values and the opinion on work in the medical field, work relationships and public expectations was administered to 374 doctors attending Continuing Medical Education courses.

**Results:**

Two personality dimensions were identified: the first dimension (which we termed "Performance Attainment") is associated preeminently with values of competence, advocacy, confidentiality, spirit of enquiry, integrity, responsibility and commitment; the second dimension (which we called “Personal Involvement”) focuses on concern and compassion. The doctors that have more difficulty accepting judgements on their activity are those who think that “Performance attainment” is less important (β = 6.01; p-value = 0.007). Instead, the doctors who believe “public expectation of the health system” is not high enough, tend to think that “Performance Attainment” is more important (β = -6.08; p-value = 0.024). The less importance is given to the values of "Personal Involvement", the less is the doctor’s perception of having a leading role in respect to other health professionals (β = -2.37; p-value = 0.018).

**Conclusions:**

Our results demonstrate that there are two different attitudes in terms of recognition and selection of the essential values to better practice the medical profession. Whether the doctors attach more importance to one dimension or the other, they do not differ in our analysis for how they answered the questions about relationships with patients, colleagues or family commitments in the questionnaire, even if they work in different areas. This suggests that in our research there is no single personal attitude that characterizes “a good doctor”.

## Introduction

There is much research on how medical professionalism can be adjusted to healthcare and social structural changes that require doctors of the 21^st^ century to modify their behaviour. The difficulty for doctors to live up to patients’ expectations of their role reflects the challenges posed by a new emphasis on the "humanization of medicine" [1] and on the figure of the "good doctor” [2]. These matters can take the forms of a dualism between doctors’ technical expertise and expectations that they empathize with the suffering of patients (Technicism vs. Humanism) [3]. In addition to this, the healthcare structures of most developed countries take into account the expectations of the "paying third party" in these already complex relationships.

Doctors’ initial training, their areas of specialisation and their ongoing education have important effects on their professional experiences. Other key influences include their work settings and length of service, but also their family environments and their lives outside the workplace.

There are new challenges in the medical field of the third millennium and the main medical associations need to work on redefining their values, limits and roles. In the 1980s a special subcommittee of the American Board of Internal Medicine (ABIM) (Subcommittee on Evaluation of Humanistic Qualities of the Internist) issued a statement that excellence in practice requires the internist to meet high standards of humanistic behaviour. This subcommittee identified integrity, respect and compassion as humanistic qualities essential for clinical competence.

In 1990, the ABIM established a project to enhance the evaluation of professionalism as a component of clinical competence and to promote the global approach of internal medicine. This project, called Project Professionalism, defined the core of professionalism as constituting those attitudes and behaviours that serve to maintain patient interest above physician self-interest. Accordingly, professionalism, as the Board has defined it within the project’s framework, aspires to altruism, accountability, excellence, duty, service, honour, integrity and respect for others [4].

In 2002 the ABIM, together with the American College of Physicians (ACP) and the European Federation of Internal Medicine (EFIM), published the “Charter of Medical Professionalism for the third millennium" [5] which identified three fundamental principles: the principle of primacy of patient welfare, the principle of patient autonomy and the principle of social justice. Despite many duties toward patients, the Charter confirmed the necessity for doctors to act in order to guarantee both the quality of care and its equitable distribution. This requires making use of all the tools and managerial methodologies they have access to and, if lacking, eventually contributing to their development.

Along this process of reconfigured medical practice there is the proposal formulated in 2005 by the Royal College of Physicians of London (RCP) [6]. It envisages that doctors show interest in their relationships with patients, colleagues, other health personnel and society as a whole. Moreover, it refers explicitly to the behavioural tendencies of the past that must be abandoned: autonomy, dominance and professional privilege. Therefore, along with redefinitions of medical professionalism, the relational aspect of the medical practice has a central role in the achievement of a fundamental principle of this profession: the ethic of care in medicine which nowadays means patient care management consisting in the centrality of the patient within a multi-professional healthcare organization [7].

Medical professionals’ attitudes to patient care management can diverge according to different personal qualities and to external circumstances, such as being part of a complex occupational health organization and the ability to balance a demanding professional life with family and home [8, 9]. In 2002 an editorial published in the BMJ emphasized the attributes of medical role models characterized by compassion towards patients and integrity, noting on the basis of interviews with medical students that not all of their Consultants had positive attitudes towards them and the patients; this may be related to the fact that not all doctors share the need for adaptation to new complex social-health organizations and of doctor-patient relationships no longer based on a paternalistic model [10].

The British Medical Association (BMA) cohort study of medical students who graduated in 2006, a 10-year longitudinal study of the career paths of 430 doctors, used a written questionnaire to evaluate the importance given by doctors to professional attitudes, in order to support medical practice and to train new professionals [11].

In our study, we adapted that questionnaire with the aim of assessing whether:

- different medical personalities exist among the medical doctors of our sample, based on the professional values to which they attributed more importance;
- personality dimensions are linked to different opinions on the core values of the profession, work relationships and public expectation;
- there is a difference in the role that doctors are conceived to play between the respondents who emphasized moral characteristics and those who identified more with values based on technical expertise.

## Methods

### Design and instruments

An anonymous self-administered questionnaire on the perception of professional values was given to doctors while attending Continuing Medical Education (CME) courses, over a five-month period, organized by the Medical Association of the Province of Padua.

The questionnaire used was derived from a translation process which included forward translation to the Italian language and back translation of two questionnaires previously used in the UK and USA [12, 13]. The Italian version of the questionnaire is available in S1 File.

The validity of the content was preliminarily evaluated according to criteria of clarity, comprehensiveness and adaptability to the national context by a working group composed of a philosopher, sociologist as well as academic and clinical doctors. This new tool was created adding to and modifying some items from the originals and the dimension of the range and the degree of association to each item was determined with respect to the attribute of interest.

A convenience sampling method was employed. The survey participation was requested by the Padua Medical Council that also obtained the participants’ consent. According to the local Ethics Committee, observational studies using completely anonymous data do not require formal approval by the Ethics Committee.

The first section of the questionnaire asked to rank from 1 (most important) to 9 (least important) nine professional qualities (concern, compassion, competence, advocacy, confidentiality, spirit of enquiry, integrity, responsibility and commitment) in order to identify different personality types. The second section explored characteristics such as “the core values of the medical profession (Q2, Q4, Q6)”, “work relationships (Q3, Q5)” and public expectations (Q7, Q8)”, through the following seven multiple choice questions (categorical ordinal variables whose answers have been placed randomly in the questionnaire to avoid a selection bias):

Q2) Commitment involved in being a doctor (from 1 = “Medicine is a vocation” to 5 = “practicing medicine is just as any other job with fixed working hours”; i.e. the higher the score the less the involvement);

Q3) Patient/doctor relationship (from 1 = “the relationship should be based on trust” to 6 = “patients are consumers of health services”; i.e. the higher the score the more formal the relationship is);

Q4) Professional regulation and complaints (from 1 = “Doctors have the duty to practice with competence” to 4 = “complaints against a doctor should be balanced between professional and lay interests”; i.e. the higher the score the less the doctor accepts judgement by others);

Q5) Teamwork and skill mix (1 = “Multidisciplinary leadership should be given to the most appropriate professional—not necessarily a doctor” to 6 = “doctors work most effectively autonomously”; i.e. the higher the score the lower the perception of the preeminence of other health professionals);

Q6) Clinical autonomy (1 = “Doctors continue to need medical training” to 4 = “Clinical freedom is important but doctors also have the responsibility to use resources effectively”; i.e. the higher the score the higher is the demand of clinical autonomy);

Q7) Public expectations about the National Health System (“too high”, “about right”, “not high enough”);

Q8) Public expectations about doctors and Medicine (“too high”, “about right”, “not high enough”).

### Statistical analysis

The comparisons of the mean ranking scores of the nine professional qualities across selected characteristics were performed, prior checking the homoscedasticity assumption, with the General Linear Model using Scheffé’s method for multiple comparisons.

Then, exploratory factor analysis was performed on the professional qualities using the principal component method, and communalities were estimated using the squared multiple correlation (SMC) approach. Criteria to determine the optimal number of factors to extract were: plot of the eigenvalues against the corresponding factor (scree plot), percentage of the common variance explained by successive factors, and the analysis of residuals. An orthogonal rotation method (Varimax) was applied to simplify the factor structure and to achieve an interpretable solution. The scores of the identified factors were rescaled between 0 (best score) and 100 (worst score), i.e. the higher the score the less the importance.

Then, the doctors’ opinions on the core values of the profession, work relationships and public expectation were analysed by selected doctors’ characteristics (gender, age group, years of practice, work area and setting). The association between these categorical variables has been assessed by the Chi-square test or Fisher exact test whenever appropriate.

Lastly, the doctors’ opinions on the core values of the profession, work relationships and public expectation were included as independent variables in multivariable linear regression models with the scores identified from the factorial analysis as dependent variables.

Software SAS v9.4 was used for the analysis.

## Results

The questionnaire was completed by 374 physicians; women represented 48% of the participants, mean age was 48.1 years (SD = 11.2; range 24–80), and the average length of medical practice was 20.7 years (SD = 11.4; range 1–55). Sixty-two percent of the doctors worked in urban centres, 25.9% in suburban areas and 11.6% in rural areas. Twenty-six percent were general practitioners (GPs), 22.3% were hospital doctors, 37.1% were specialists working in the private sector, 5.4% were academic doctors, 3.8% worked in health service and management, and 5.1% dealt with continuity of care.

Table 1 shows the importance ascribed by doctors to different professional values, overall and by selected characteristics. Competence is the medical attitude recognized as the most important, with an average ranking score of 3.1, and responsibility comes second (mean 3.5). It is noteworthy that compassion and concern are the lowest rated professional values (mean values are 5.4 and 5.2, respectively). Statistical difference is only found for gender where male doctors give more importance to competence (3.6 versus 2.6), advocacy (4.9 versus 3.9) and responsibility (3.9 versus 3.1).

**Table 1:** Mean values of the ranking scores of the nine professional qualities overall and across sex, age, years of practice, work area and setting.

	Concern	Compassion	Competence	Advocacy	Confidentiality	Spirit of enquiry	Integrity	Responsibility	Commitment
Overall	5.2	5.4	3.1	4.4	4.9	4.4	4.2	3.5	4.1
Gender									
Male	5.2	5.2	3.6	4.9	5.0	4.5	4.2	3.9	4.3
Female	5.2	5.6	2.6	3.9	4.7	4.2	4.3	3.1	3.9
*p-value*	*ns*	*ns*	*0*.*002*	*0*.*001*	*ns*	*ns*	*ns*	*0*.*006*	*ns*
Age									
24–29	5.0	5.3	3.1	4.3	4.7	4.2	3.7	3.1	4.3
30–49	4.9	5.3	2.9	4.1	4.8	3.9	4.3	3.3	4.1
50–69	5.4	5.5	3.2	4.6	4.9	4.6	4.3	3.6	4.0
70–80	5.8	5.7	5.3	6.0	5.1	5.6	6.1	5.7	6.4
*p-value*	*ns*	*ns*	*ns*	*ns*	*ns*	*ns*	*ns*	*ns*	*ns*
Years of practice									
0–10	4.9	5.3	2.9	4.0	4.6	4.0	4.0	3.2	4.0
11–30	5.3	5.6	3.2	4.6	5.0	4.4	4.3	3.6	4.1
31–50	5.2	5.1	3.0	4.7	5.0	4.8	4.3	3.7	4.2
*p-value*	*ns*	*ns*	*ns*	*ns*	*ns*	*ns*	*ns*	*ns*	*ns*
Work area									
Urban	5.4	5.5	3.0	4.4	4.9	4.4	4.2	3.4	4.0
Suburban	4.7	5.2	3.1	4.3	4.7	4.3	4.3	3.6	4.2
Rural	5.0	5.3	3.8	4.6	4.8	4.4	4.6	4.0	4.6
*p-value*	*ns*	*ns*	*ns*	*ns*	*ns*	*ns*	*ns*	*ns*	*ns*
Work setting									
GP, Private, Continuity of Care	5.1	5.3	3.3	4.7	5.1	4.6	4.4	3.9	4.4
Hospital, Teaching hospital	5.3	5.4	3.0	4.5	5.1	4.3	4.3	3.2	4.3
Service and management	5.2	5.5	3.0	4.1	4.4	4.2	4.0	3.3	3.7
*p-value*	*ns*	*ns*	*ns*	*ns*	*ns*	*ns*	*ns*	*ns*	*ns*

ns = not significant (p-value>0.05).

The exploratory factor analysis applied to the nine professional values identified a two-factor solution. Table 2 shows the estimated rotated factor loadings of the two identified factors. The values of competence, advocacy, confidentiality, spirit of enquiry, integrity, responsibility and commitment load highly on Factor 1 and have lower or negligible loadings on Factor 2; since these items measure the degree to which technical expertise is considered important, we called this personality dimension as “Performance attainment”. On the other hand, the values concern and compassion load highly on Factor 2 and have negligible loadings on Factor 1; in this case the personality dimension was called “Personal involvement”.

**Table 2:** Estimated rotated factors loadings of the two personality dimensions.

	Concern	Compassion	Competence	Advocacy	Confidentiality	Spirit of enquiry	Integrity	Responsibility	Commitment
Factor 1: Performance attainment	0.17	-0.002	**0.83**	**0.57**	**0.61**	**0.75**	**0.73**	**0.93**	**0.79**
Factor 2: Personal involvement	**0.71**	**0.65**	-0.09	0.43	0.55	0.39	0.22	0.03	0.29

Table 3 shows the distribution of the answers to the questions Q2-Q8 by gender, age, years of practice, work area and setting. Significant associations have been found between (i) the patient/doctor relationship (Q3) and the work setting; (ii) professional regulation and complaints (Q4) with gender and work setting; (iii) teamwork and skill mix (Q5) with age and years of practice; (iv) clinical autonomy (Q6) with years of practice and work setting; and (v) public expectations (Q7) and work setting.

**Table 3:** Distribution (%) of the Q2-Q8 questions by gender, age, years of practice, work area and setting.

		Overall	Gender			Age group					Years of practice				Work area				Work setting			
			Male	Female	p-value	24–29	30–49	50–65	66–80	p-value	0–10	11–30	31–55	p-value	Urban	Sub-urban	Rural	p-value	GP, Private, Continuity of Care	Hospital, Teaching hospital	Service and management	p-value
**Q2**	**a)** **b)** **c)** **d)** **e)**	7.3 7.0 29.3 56.2 0.2	8.8 7.3 33.8 50.0 0.0	5.6 6.7 24.5 62.5 0.6	ns	5.7 2.8 31.4 60.0 0.0	4.7 4.7 22.2 67.4 0.8	8.8 8.8 32.5 49.7 0.0	14.3 14.3 57.1 14.3 0.0	ns	4.3 6.5 27.1 62.0 0.0	8.6 7.2 28.7 55.0 0.5	7.1 7.1 34.3 51.4 0.0	ns	5.6 6.5 32.4 55.4 0.0	12.6 7.3 25.2 53.7 1.0	4.6 9.3 23.2 62.8 0.0	ns	6.1 5.3 29.5 59.1 0.0	7.7 5.8 34.9 51.4 0.0	8.1 9.6 24.4 57.0 0.7	ns
**Q3**	**a)** **b)** **c)** **d)** **e)** **f)**	49.3 36.2 1.9 9.4 1.3 1.9	50.2 34.7 2.0 10.3 1.0 1.5	48.6 37.4 1.7 8.3 1.7 2.2	ns	42.8 40.0 0.0 17.1 0.0 0.0	50.4 38.5 0.8 8.6 0.8 0.8	50.2 33.0 2.9 8.8 2.0 2.9	42.8 57.1 0.0 0.0 0.0 0.0	ns	51.1 35.8 0.0 11.9 1.1 0.0	47.1 39.0 2.8 8.5 0.5 1.9	54.3 27.1 1.4 8.5 4.3 4.3	ns	48.9 34.6 1.3 11.2 1.7 2.1	54.1 35.4 1.0 7.3 1.0 1.0	41.8 44.2 7.0 4.6 0.0 2.3	ns	49.2 34.1 4.5 6.8 1.5 3.8	58.2 29.1 0.0 12.6 0.0 0.0	43.3 43.3 0.0 9.5 2.2 1.5	0.0046
**Q4**	**a)** **b)** **c)** **d)**	82.8 1.3 15.3 0.5	78.4 2.6 18.6 0.5	88.2 0.0 11.2 0.5	0.0102	80.0 0.0 20.0 0.0	86.6 0.8 12.6 0.0	81.7 2.0 15.7 0.5	71.4 0.0 14.3 14.3	ns	86.9 1.1 10.8 1.1	81.9 1.4 16.2 0.5	81.4 1.4 17.1 0.0	ns	83.1 1.3 15.1 0.4	81.2 1.0 17.7 0.0	86.0 2.3 9.3 2.3	ns	76.5 0.7 22.7 0.0	91.2 2.9 5.8 0.0	83.1 0.7 14.7 1.4	0.0013
**Q5**	**a)** **b)** **c)** **d)** **e)** **f)**	20.6 31.6 16.1 14.2 16.1 1.3	18.1 30.0 18.6 12.9 18.6 1.5	23.4 33.5 13.4 15.0 13.4 1.1	ns	37.1 28.5 5.7 20.0 8.5 0.0	30.7 28.3 14.9 7.9 15.7 2.3	11.3 35.0 19.2 17.2 16.7 0.5	28.5 14.3 0.0 0.0 42.8 14.3	<0.0001	33.7 27.1 13.0 13.0 10.8 2.1	14.7 31.9 20.4 13.8 17.6 1.4	21.4 37.1 7.1 15.7 18.5 0.0	0.012	20.3 30.3 15.1 15.1 18.2 0.8	22.9 36.4 16.6 12.5 11.4 0.0	18.6 25.6 20.9 11.6 16.3 7.0	ns	21.2 32.6 18.2 14.4 12.8 0.7	25.2 33.0 13.6 11.6 16.5 0.0	16.9 30.1 16.1 15.4 18.3 2.9	ns
**Q6**	**a)** **b)** **c)** **d)**	53.5 14.5 10.5 21.5	51.3 18.3 11.4 19.1	56.2 10.6 9.0 24.1	ns	62.8 11.4 8.5 17.1	59.0 13.4 7.8 19.7	48.5 15.3 11.9 24.2	57.1 28.5 14.3 0.0	ns	63.0 8.7 8.7 19.6	51.9 13.3 10.4 24.3	46.3 26.1 11.6 15.9	0.039	54.3 14.7 11.7 19.1	56.2 11.4 9.3 22.9	41.8 20.9 4.6 32.5	ns	42.4 17.4 9.1 31.0	62.1 11.6 9.7 16.5	57.7 14.0 11.8 16.3	0.018
**Q7**	**a)** **b)** **c)**	53.1 38.1 8.8	52.3 40.4 7.2	54.2 35.2 10.6	ns	51.4 42.9 5.7	54.3 35.4 10.2	54.2 36.9 8.9	14.3 85.7 0.0	ns	50.0 41.7 8.3	54.0 37.4 8.6	54.4 35.5 10.0	ns	49.8 40.7 9.5	54.2 34.4 11.4	67.4 32.6 0.0	ns	63.6 27.3 9.1	53.4 41.8 4.8	43.4 44.8 11.8	0.006
**Q8**	**a)** **b)** **c)**	65.2 28.4 6.4	64.2 30.1 5.7	66.5 26.3 7.3	ns	74.3 20.0 5.7	65.3 29.9 4.7	65.0 27.1 7.9	28.6 71.4 0.0	ns	69.1 26.2 4.7	64.6 27.8 7.6	63.3 31.1 5.5	ns	63.6 29.0 7.4	65.6 27.1 7.3	72.1 27.9 0.0	ns	73.5 21.2 5.3	64.1 30.1 5.8	58.1 33.8 8.1	ns

Key: Q2) Commitment involved in being a doctor; Q3) Patient/doctor relationship; Q4) Professional regulation and complaints; Q5) Teamwork and skill mix; Q6) Clinical autonomy; Q7) Public expectations about the National Health System; Q8) Public expectations about doctors and medicine.

The seven questions were then included as predictors in the multivariable regressions on the two personality dimensions scores; the results are shown in Table 4.

**Table 4:** Multivariable analysis of the two factors in the seven questions.

Questions	Performance attainment		Personal Involvement	
	Parameter (SE)	p-value	Parameter (SE)	p-value
**Q4**	6.01 (5.2)	0.007		
**Q5**			-2.37 (1.0)	0.018
**Q7**	-6.08 (2.7)	0.024		

Q4) Professional regulation and complaints; Q5) Teamwork and skill mix; Q7) Public expectations about the National Health System; Q8) Public expectations about doctors and medicine.

The effects of questions Q4 and Q7 on “Performance attainment” are statistically significant: the positive parameter of Q4 (6.01) indicates that the doctors that have more difficulty accepting judgments on their activity are those who think that “Performance attainment” is less important. Instead, the negative parameter of Q7 (-6.08) indicates that the doctors who believe “public expectation of the health system” is not high enough, tend to think that “Performance attainment” is more important.

In the regression model for the “Personal involvement” score, only question Q5 has a statistically significant effect which is negative (-2.37) indicating that the less importance is given to the values of concern and compassion, the less is the doctor’s perception of having a leading role respect to other health professionals (β = -2.37; p-value = 0.018).

## Discussion

Our study, based on a questionnaire self-administered to physicians, aimed to assess the core values of medical professionals and their attitudes towards critical and complex issues related to being a doctor in the 21st century. In considering the choice of values linked to the medical profession, we also explored whether different personality dimensions emerged between the participants and if, in turn, these different personalities were associated with different attitudes towards work in the medical field.

Regarding the importance given to professional values, the respondents chose in descending order: competence (more than half of the respondents ascribed the greatest importance to it), responsibility, integrity, commitment, and then advocacy, confidentiality, spirit of enquiry, concern and, lastly, compassion. Professional competence represents the “cardinal value” of our sample, confirming the results obtained in a previous study [14].

On the other hand, the respondents gave the least importance to compassion. The low score given to the value of compassion could be interpreted with the help of a recent study [15]. In general terms it is difficult to distinguish compassion from “care”. However, if one accepts the etymological Latin significance of “cum patio” (that indicates a sentiment for which a person emotionally perceives the suffering of others), one can understand how work overload could limit professionals in their approach to patients and their family members with the right attention. This difficulty is a major cause of reduced ability to be compassionate, which could lead to the psychological disorder called compassion-fatigue [16].

The second part of the questionnaire concerned aspects of the respondents’ professional lives. Regarding their levels of commitment to being a doctor, about 60% of the sample expressed their right to have enough time to enjoy home life. This confirms the BMA survey’s result: according to the English physicians interviewed by the BMA, one of the most important factors affecting the medical profession is the need to balance workloads with family life [11]. In recent years, the demand and desire to obtain part-time posts has increased, especially among general practitioners, and this has led to the request to develop more flexible types of employment contracts [17]. In Italy, this data is confirmed by a recent survey conducted by a national physician syndicate (Anaao Assomed) [18].

Regarding the doctor-patient relationship, our data show that the work setting influences this connection.

Hospital physicians ascribe more importance to mutual trust in the doctor-patient relationship than general practitioners do, and tend to be more involved in issues regarding professional responsibility,

This difference can be discerned better in national public health systems (like the Italian one) in which the GP has the role of discerning patients in need of referral to a medical specialist or of hospitalization, while the hospital specialist is the ultimate professional figure for the care of acute and/or complex medical cases.

Clinical autonomy is considered a value to be defended and continuing medical education is an essential duty.

Other significant data regard professional regulation and compliance, and the relationships with other professional figures. Male doctors, more often than female, consider that the control of medical skills regards the medical class (the only one capable of judging adequately). Concerning the relationships with other professional figures, the doctors with less years of practice have a higher propensity to think that only physicians should supervise the management of health services. Surprisingly, younger doctors tend to preserve the leadership of physicians relative to other professional health categories, perhaps defending a role they have recently come to hold.

The literature shows that sometimes the skill and competence of doctors are accompanied by inappropriate behaviour, such as impatience and arrogance in relationships with patients and colleagues. Thus, aspects of a “bad doctor” and those of a “good doctor” can coexist in the same person; Gwen Adshead, a forensic psychotherapist retains that disruptive clinicians were often “clinically excellent.” [19]. For this reason, we have evaluated whether the doctors’ viewpoints over some professional values correspond to a different way of perceiving work and relationships with patients, colleagues and other health professionals. The factor analysis identified two dimensions of doctors’ personality. Competence, responsibility and commitment are the values that demarcate the first dimension, while concern and compassion are the two distinctive values of the second personality dimension.

The existence of two separate dimensions is in accordance with a previous study conducted in an Irish medical school sample that showed low correlations between empathy and professionalism measures [20]. Montgomery, in a systematic review of the literature to define humanism in the practice of medicine, uses a model centred on the interaction between heart and mind in which the heart represents the emotive domains of empathy, compassion and connectedness while the head represents the cognitive domains of knowledge [21]. In medicine, these often are described under the rubric of professionalism and under the rubrics of ethics and epistemology.

Therefore, our work based on answers given and values recognized as important by the participants seems to characterize a duplicity of attitudes that, for the close interconnection between professionalism and humanism, we could call “Performance attainment” for the first group of values and “Personal involvement” for the second.

Regarding teamwork, the professionals with a high “Personal Involvement” score tend to believe that the doctor in a multidisciplinary team has an equal weight and value compared to other professional figures. Concerning professional competence and regulation, the professionals with a high “Performance attainment” score believe in the necessity of technical expertise and the need to undergo verification. By contrast, professionals with a propensity for the “Personal Involvement” dimension do not consider these to be compelling issues. The tendency for the two identified personality dimensions does not seem to be associated with the medical duties and the availability of extra working time, the doctor-patient relationship and clinical autonomy.

Campbell studied the degree to which UK doctors agreed with the norms of professionalism contained in "the Charter". He also explored reasons for a discrepancy between a high level of agreement with the rules and a frequent behaviour not compliant with these in the performance of professional activity [13]. In our study, adherence to norms was not evaluated. However, we studied whether the two personality dimensions, emerging from the scores given to professional values, showed different aptitudes towards some critical aspects of relationships, which represent how professionalism expresses itself and “reflect the multifaceted nature of this fundamental aspect of being a physician and practicing medicine” [22].

Healthcare of patients and competence are the characteristics of two non-dichotomous but interpenetrated aspects called "Professionalism" and "Humanism". Professionalism is defined as a "way of acting" which includes a set of well-coded behaviours. According to Cohen, a doctor could play his/her professional role as a sort of duty, without believing in the principles that underlie it and without sharing its motivations [3]. On the contrary, so-called Humanism is a "way of being" that includes a series of deep convictions concerning the conception of the sense of duty towards others, especially if in distress. Humanism manifests itself through personal characteristics, such as altruism, responsibility, integrity, respect for others and compassion. Our work based on the responses given on a self-administered questionnaire seems to support, through the factor analysis, the presence of these two dimensions of medical sentiment and the doctors that characterize themselves differently also respond differently to selected professional attitudes, such as professional regulation and complaints (Q4), public expectations about the National Health System (Q7) and team-work and skill mix (Q5).

In conducting our work we made two choices that may represent interpretative limits for the results. We decided to use the questionnaire applied in a previous survey on professional values by the BMA. It is noteworthy that the translation of the term “confidentiality”, which in addition to referring to one of the higher Hippocratic values, in Italian it is a term that lends itself to various meanings. It should be emphasized, however, that the qualities necessary to effectively manage the doctor-patient relationship cannot be reduced to a single concept such as this.

Linguistic sensitivity in understanding the terminology of the questionnaire is not the only regional factor that can influence the generalization of our results. It has been shown that the difference in countries’ economies and in the organizational structure of national health systems can influence the professional conduct of doctors and dentists even regarding the perception of certain ethical and moral values [23].

A limitation also found in other surveys [24] is that self-reported behaviours could have been biased by the social impression the respondent wished to give, even when we tried to protect the anonymity of the questionnaires. Furthermore, in characterizing the attitudes of the doctors participating in the study, we analyzed the responses to questions and not the observation of the respondents’ behaviour. However, it is noteworthy that the expression of an opinion by these doctors may represent an expression of a professional role model in which they can identify themselves.

Moreover, the aim of our study could lend itself more to a qualitative analysis because it is difficult to measure the values of a “good doctor” with quantitative methods. However, obtaining statistical insights into qualitative problems seemed to us an important result and the more relevant strengths of our work were to statistically demonstrate the aforementioned conceptual dualism of medical professionalism.

Being a "professional" (from the Latin "professio" that indicates a public oath of loyalty) implies a fundamental public value, a commitment, a pact established with the community that should be based on the application of ethical virtues. By focusing on the type of person that he/she should be—a moral agent, a man with such characteristics: the Aristotelian virtues–a professional can do his job well as a virtuous doctor, and be able to pursue the best interest of the patient [22].

Our data seem to confirm that, in terms of recognition and selection of the essential values to better practice the medical profession, there are two attitudes. These two personality dimensions, however, do not differ in how doctors value their relationships with patients and colleagues, even if they work in different settings. In other words, our results suggest that a “good” doctor cannot be entirely subsumed into either category.

## Supporting information

S1 File Italian version of the administered questionnaire. (PDF)
