# Supplementary material for: Survey of doctors’ perception of professional values
Source: PLoS One. 2020 Dec 28;15(12):e0244303. doi: 10.1371/journal.pone.0244303 (PMC7769469; doi:10.1371/journal.pone.0244303)
Supplement: S1 File — (PDF) [file pone.0244303.s001.pdf]

QUESTIONARIO SUI VALORI DELLA PROFESSIONE MEDICA

Caro collega,

il medico dei giorni nostri è chiamato ad operare efficacemente ed efficientemente in un contesto turbolento e la relazione col paziente si modifica mano a mano che le informazioni sanitarie diventano sempre più facilmente disponibili. La professione medica è sempre più in discussione.
Un gruppo di lavoro composto da medici operanti nei diversi settori della sanità (territorio, ospedale e università) ha elaborato il presente questionario che aiuterà ad affrontare questi temi nel Convegno annuale dell'Ordine dei Medici di Padova che si terrà nel Novembre 2010.
Ti prego pertanto di voler collaborare esprimendo le tue opinioni sui valori professionali del medico d'oggi contrassegnando un'unica risposta.

Il gruppo dell'Ordine dei Medici di Padova "Pensare per la professione"

Sesso M ☐ F ☐ Età [......] N. anni di laurea [......]
La tua sede di lavoro principale è in un centro: urbano ☐ suburbano ☐ rurale ☐
Qual è il tuo ambito di lavoro?
☐ Medicina Generale ☐ Pediatria di Libera Scelta ☐ Continuità Assistenziale ☐ Ospedale
☐ Università ☐ Medico di organizzazione ☐ Altro, specificare..

1) Ordina questi valori professionali secondo l'ordine di importanza (da 1 il più importante a 9 il meno importante) che tu ritieni abbiano per un medico

VALORI	1	2	3	4	5	6	7	8	9
**Affezione**									
**Compassione**									
**Competenza**									
**Difesa della causa della salute (advocacy)**									
**Discrezione**									
**Indole all'approfondimento**									
**Integrità morale**									
**Responsabilità**									
**Senso del dovere**									

2) Esprimi il tuo giudizio su quale dovrebbe essere il livello di coinvolgimento personale nella professione medica segnando una sola delle seguenti frasi

☐ esercitare la medicina è un impegno importante, e il medico non dovrebbe attendersi un modello di vita privata uguale a coloro che non sono medici
☐ esercitare la medicina è un impegno importante ma il medico deve avere anche una vita familiare decente e dei tempi per lo svago
☐ essere medico è diventato un lavoro come un altro e i medici hanno diritto ad un orario di lavoro normale e di dimenticarsi del lavoro quando sono a casa
☐ la medicina è una vocazione e solo coloro che sono preparati a farne il loro impegno primario dovrebbero esercitare la medicina
☐ l'esercizio della professione medica dovrebbe essere organizzato in un modo che permetta al medico di bilanciare impegno lavorativo, carriera, famiglia e altri interessi

3) Esprimi il tuo parere su quale frase sintetizzi meglio il rapporto medico-paziente segnando una delle seguenti frasi

☐ Esistono circostanze in cui un medico può decidere che è nell'interesse del paziente non fornirgli tutte le informazioni sulla sua patologia

- ☐ Il medico dovrebbe fornire al paziente tutte le informazioni disponibili sulla sua malattia e permettere che il paziente compia le proprie scelte sui trattamenti a cui deve sottoporsi
- ☐ I medici fanno parte di un contesto sanitario problematico poiché sono stati formati incoraggiando la selezione delle informazioni da dare ai pazienti per cui ai pazienti dovrebbe essere consentito di ricorrere ad un consulente esterno se lo desiderano
- ☐ I pazienti sono oggi dei clienti del sistema sanitario e i medici devono rispondere alle loro domande
- ☐ La relazione medico-paziente dovrebbe essere un rapporto basato su reciproca fiducia e disponibilità
- ☐ La relazione medico-paziente non può mai essere paritaria perché le conoscenze che il medico possiede lo pongono sempre in una posizione di superiorità

4) Esprimi il tuo parere sulle competenze mediche e sugli aspetti del loro controllo

- ☐ I medici hanno il dovere di assicurare che lavorano con esperienza e preparazione e la loro competenza deve evolvere attraverso la formazione continua, gli incontri clinici e periodiche verifiche formali delle loro capacità
- ☐ I medici non sono solo responsabili delle loro azioni ma anche in maniera collegiale delle azioni dei loro colleghi. Essi dovrebbero essere pronti a deferire un collega che non raggiunge gli standard professionali richiesti
- ☐ Quando un'accusa è rivolta a un medico è nel migliore interesse del paziente che sia preso in considerazione il rapporto esistente tra professione ed interessi legali
- ☐ Solo i medici hanno le competenze e l'esperienza necessaria a giudicare le azioni dei loro pari quando sono poste delle critiche sul loro operato

5) Esprimi il tuo parere su questi aspetti riguardanti il rapporto con altre figure professionali

- ☐ Il tradizionale ruolo e compiti del medico devono essere protetti contro la loro frammentazione con altre figure professionali
- ☐ I medici come professionisti sanitari maggiormente preparati dovrebbero essere in grado di gestire i servizi sanitari
- ☐ I medici sono i professionisti sanitari maggiormente preparati e conseguentemente sono i naturali leaders di equipe multi professionali
- ☐ I medici lavorano più efficacemente come autonomi professionisti
- ☐ La direzione di un approccio multidisciplinare alle cure individuali o a gruppi di pazienti deve essere affidata al professionista più adatto – non necessariamente un medico
- ☐ Le opinioni di tutti i membri di un team multidisciplinare di professionisti sanitari hanno uguale valore e peso

6) Esprimi il tuo parere sull'autonomia clinica del medico

- ☐ I medici necessitano di una vasta formazione per mantenersi costantemente innovativi e flessibili e in grado di aggiornarsi durante tutta la loro carriera
- ☐ Lavorare in accordo con linee guida che evidenziano la migliore pratica è accettabile se sono sviluppate per la professione e il loro non rispetto non costituisce riscontro penale
- ☐ L'esercizio dell'indipendenza clinica è importante, ma i medici hanno anche il dovere di usare le risorse efficacemente
- ☐ L'indipendenza clinica è essenziale e i medici devono combattere contro chi vuole limitarla

7) Ritieni che le aspettative pubbliche verso quello che il sistema sanitario può fare siano

☐ troppo alte ☐ giuste ☐ non sufficientemente elevate

8) Ritieni che le aspettative pubbliche verso quello che i medici possono fare siano

☐ troppo alte ☐ giuste ☐ non sufficientemente elevate

GRAZIE!

Le domande sono tratte da un'indagine della British Medical Association, 2005, relativa alla BMA Cohort Study of 1995 Graduates

Il questionario è scaricabile dal sito dell'Ordine www.omco.pd.it
